# Investigation into fatigue micro-crack identification of steel bridge decks based on acoustic emission detection technology

**DOI:** 10.1371/journal.pone.0317969

**Published:** 2025-04-29

**Authors:** Li Jiaqing, Song Fei, Xiao Zidong, Zhu Longji, Chen Lan, Wei Zheliang

**Affiliations:** 1 Guangdong Shengxiang Traffic Engineering Testing Co. Ltd., Guangzhou, China; 2 The Fourth Company of China Construction Sixth Engineering Bureau Co. Ltd., Shenzhen, China; 3 Guangdong Jiaoyue Engineering Testing Co. Ltd., Guangzhou, China; 4 School of Civil Engineering and Surveying & Mapping Engineering, Jiangxi University of Science and Technology, Ganzhou, China; 5 Heyuan Polytechnic, Heyuan, China; 6 Zhejiang Academy of Surveying and Mapping, Hangzhou, China; Politecnico di Torino, ITALY

## Abstract

With increasing traffic loads and extended bridge service life, fatigue damage in steel bridge decks has become a significant concern. Traditional detection methods often lack the accuracy and responsiveness needed for practical engineering applications. To address the non-stationary nature of acoustic emission (AE) signals during crack initiation and propagation, this study combines the K-singular value decomposition (K-SVD) dictionary learning algorithm with convolutional neural networks (CNN) to enhance AE signal processing and fatigue crack detection. The K-SVD algorithm functions as an adaptive filter, learning from AE signals in various damage states to remove background noise and retain critical structural characteristics. This processed AE data is then input into a CNN, where the improved signal clarity enables higher classification accuracy. Specifically, the integration of K-SVD with CNN achieved recognition accuracies of 93.64% and 92.56% for AE signals from damaged areas, and 95.32% and 94.27% for undamaged signals, on training and test sets, respectively. This approach demonstrates strong engineering potential by providing a scalable solution for real-time, accurate crack detection in bridge inspections. Though computationally intensive, K-SVD’s adaptive dictionary learning enhances CNN performance, making the combination viable with optimization strategies in practical settings. These results provide a theoretical foundation and practical guidance for improving fatigue crack detection in steel bridge decks, supporting future applications in automated bridge inspection.

## 1. Instruction

With the rapid development of modern transportation, steel structure bridges have gained popularity due to their lightweight, strong crossing capacity, and high construction cycle utilization rate [1–4]. They are widely used in the construction of modern highways, railways, and urban road bridges [5–7]. Among these, orthotropic steel bridge decks are indispensable for large-span bridge structures. Their advantages include good integrity, strong bearing capacity, and convenient construction, making them one of the significant innovations in modern bridge engineering [8–10]. However, steel bridge decks are prone to fatigue micro-cracks during long-term service due to repeated loads, corrosion, and fatigue. The initiation and propagation of these micro-cracks not only reduce the structural safety and service life of steel bridges but may also lead to catastrophic accidents [11,12]. Therefore, early detection and identification of micro-cracks in steel bridge decks are crucial.

Despite the critical need, effective detection of fatigue cracks in steel bridge decks remains challenging. Traditional inspection methods, such as visual inspection, ultrasonic testing, and magnetic particle inspection, often suffer from limitations including low accuracy, susceptibility to human error, and inability to provide real-time monitoring [13–15]. These limitations make it difficult to capture the subtle features of early-stage micro-cracks, often resulting in late detection only after cracks have grown to potentially dangerous sizes. As a result, current research is exploring advanced non-destructive techniques, such as AE monitoring, which can capture high-frequency signals emitted during crack initiation and growth [16–18]. However, AE signals are often non-stationary and highly susceptible to background noise, complicating the extraction of useful information related to damage.

In recent years, AE technology has become a valuable tool for real-time monitoring of structural health, especially in detecting and analyzing fatigue cracks in complex structures like steel bridge decks. AE technology’s primary advantage lies in its sensitivity to the early stages of crack initiation and propagation. Unlike traditional methods such as visual inspection, ultrasonic testing, and magnetic particle inspection, which often require significant crack development before detection is possible, AE can capture the high-frequency energy released during micro-crack formation, allowing for early-stage identification of structural deterioration [21–23]. Additionally, AE enables continuous and remote monitoring, which makes it especially suitable for large infrastructure projects where frequent manual inspections may be impractical or disruptive. AE techniques also have the benefit of detecting internal defects without requiring direct access to the damaged area, a significant advantage over methods that rely on surface-level observations. However, AE technology is not without limitations. AE signals are highly sensitive to external noise, which can complicate data interpretation, particularly in field applications where environmental noise and other operational activities may interfere. Furthermore, AE produces non-stationary signals that are challenging to analyze due to their complex, transient nature. This limitation has driven the development of advanced signal processing techniques, such as K-SVD, wavelet transforms, and empirical mode decomposition. These are used to improve AE data quality and extract meaningful features from noisy backgrounds.

To address the issue of noise in AE signals, the K-SVD dictionary learning method has been introduced into the filtering process. K-SVD algorithm is widely used in noise reduction and feature extraction in medical image processing and speech recognition [19,20]. Because it can adaptively construct a dictionary to represent the significant features of non-stationary signals. K-SVD dictionary learning is an effective signal processing technique that uses a learned dictionary to sparsely represent AE signals, thereby achieving signal denoising and feature extraction [21–24]. This method significantly improves the signal-to-noise ratio of AE signals while preserving the key features of crack signals, providing a reliable data foundation for subsequent micro-crack identification. Other advanced signal processing techniques, such as wavelet transforms, empirical mode decomposition (EMD), and sparse representation, have also been widely studied for their ability to handle non-stationary and noisy signals. For example, Inderyas et al. [25] developed a one-dimensional convolutional neural network (1D-CNN) model to filter AE data in reinforced concrete beams, achieving high classification accuracy in distinguishing true AE signals from noise-induced ones. Yang et al. [26] proposed a method using stacked denoising autoencoders for AE source localization and identification in metallic plates, demonstrating enhanced performance in noisy environments. Wavelet transforms are commonly used for multi-scale analysis and denoising in structural health monitoring, while EMD has proven effective for decomposing signals into intrinsic mode functions that reveal hidden structures [27,28]. Compared to these methods, K-SVD’s adaptive nature provides a unique advantage in real-time applications where varying damage states produce different AE signal characteristics. However, integrating K-SVD with machine learning models presents additional challenges in optimizing the signal processing chain. Studies indicate that coupling K-SVD denoising with deep learning architectures can substantially enhance classification accuracy by delivering higher-quality input data [29,30]. By building upon these insights, this study aims to leverage K-SVD’s adaptability to enhance machine learning models feature extraction, achieving a robust and efficient detection framework for real-time fatigue crack monitoring in steel bridge decks.

CNN as a deep learning model, have been widely used in fields such as image and speech recognition due to their powerful feature extraction and pattern recognition capabilities [31–33]. CNN has strong pattern recognition performance and is widely used in image classification, speech recognition and other scenes requiring highly accurate recognition [34,35]. In identifying micro-cracks in steel bridge decks, CNNs can automatically extract spatiotemporal features from a large number of AE signal samples and accurately classify and identify micro-cracks. Compared to traditional machine learning methods, CNNs have significant advantages in processing complex data and improving recognition accuracy. For instance, Li et al. [36] used characteristic mode components of empirical mode decomposition as input and established a composite material damage AE pattern recognition method based on principal component analysis and support vector machines. Li et al. [37] proposed a rail crack monitoring method based on AE signal time-frequency maps and CNN.

In summary, fatigue micro-crack identification of steel bridge decks based on AE detection technology holds important theoretical significance and engineering application value, providing new technical means for health monitoring and maintenance of steel bridges. Based on the characteristics of the CNN network, the K-SVD dictionary learning algorithm filters the noise-containing AE signals. The short-time Fourier transform is then applied to these filtered AE signals to obtain their frequency domain information. Both the time domain and frequency domain waveforms of the filtered AE signals are normalized. Singular value decomposition is performed on the processed AE signals to extract the time domain and frequency domain characteristics of damaged and undamaged steel structures, facilitating their classification. The learning and testing datasets for the CNN network are generated through these steps. The feature matrix of the AE signals, obtained from singular value decomposition, serves as the input layer. The output layer classifies undamaged and damaged steel structures. The test results of the CNN network are compared with actual results to verify the network’s accuracy. The CNN network can be trained on limited data samples to effectively identify fatigue damage cracks in steel bridge deck panels. A new high-precision method for identifying fatigue micro-cracks in steel bridge decks is proposed, offering theoretical support and technical reference for achieving efficient and accurate detection of micro-cracks in steel bridge decks.

## 2. Analysis of denoising effect of AE signals based on K-SVD dictionary learning

### 2.1 Basic principles of K-SVD dictionary learning

In contrast, K-SVD is an adaptive dictionary learning algorithm that iteratively optimizes the representation of signals by building a dictionary tailored to the specific features of the AE data. This adaptability allows K-SVD to learn basic functions that best capture the unique characteristics of AE signals under different damage states. As a result, K-SVD effectively separates noise while preserving essential structural features in AE signals, providing cleaner and more representative inputs for subsequent classification by machine learning models such as CNN. Furthermore, K-SVD has been shown to outperform fixed-basis methods in applications like image and speech processing, making it a promising choice for AE signal denoising. Dictionary learning for filtering AE signals involves two main processes. The first process is sparse encoding of AE signal data. The second process is selecting appropriate algorithms to learn and update the dictionary.

The collected AE signal data mainly includes AE signal and background noise signal, as shown below:


$$f_{1}(t)=f(t)+\varepsilon (t)$$ (1)

In the equation, f(t) represents the effective AE signal. ε(t) represents the background noise signal. $f_{1}(t)$ represents the collected AE signal. The goal of K-SVD dictionary learning is to suppress ε(t) and obtain the collected AE signal $f_{1}(t)$ that approximates f(t) signal.

#### Sparse coding.

Based on the collected AE signal features, an initial dictionary is obtained. The orthogonal matching pursuit algorithm (OMP) was used to perform sparse processing on each collected AE signal.


$${\text{min}}_{\text{D, X}}\{||f(t)-\text{DX}|{|}_{F}^{2}\}\text{s}.t\text{. }\forall i,\text{||}x_{i}|{|}_{0}\leq T$$ (2)

In the formula, ***D*** represents the dictionary to be trained. ***X*** represents the sparse coefficient matrix corresponding to the dictionary D. *x*_i_ represents the sparse coefficient vector. ***T*** represents the sparsity. *F* represents the Frobenius norm.

#### Dictionary update.

The K-SVD algorithm is used to update the dictionary. The iterative idea is used to solve ***D*** and ***X*** alternately during the update process. The essence of dictionary update is to obtain the optimal sparse coefficient matrix ***X*** and its corresponding dictionary ***D*** through multiple iterative calculations. Therefore, the constraint condition in this stage is equation (2) [30]. The K-SVD algorithm update dictionary process is as follows:


$$|f(t)-\text{DX}|{|}_{F}^{2}=||f(t)-\sum_{j=1}^{t}d_{j}x_{j}|{|}_{F}^{2}=||(f(t)-\sum_{j\neq k}^{t}d_{j}x_{j})-d_{k}x_{k}|{|}_{F}^{2}$$ (3)

In the formula, $x_{j}$ represents the *j*-th row vector of the sparse matrix ***X***. $d_{j}$ represents the *j*-th column vector of the dictionary ***D***. Defining $E_{k}$ as the computational error generated by atoms other than the *k*-th atom, the above formula can be simplified as:


$$|f(t)-\text{DX}|{|}_{F}^{2}=||E_{k}-d_{k}x_{k}|{|}_{F}^{2}$$ (4)

The error matrix is decomposed using the principle of singular value decomposition.


$$E_{k}=U\Delta V^{T}$$ (5)

**Fig 1:**
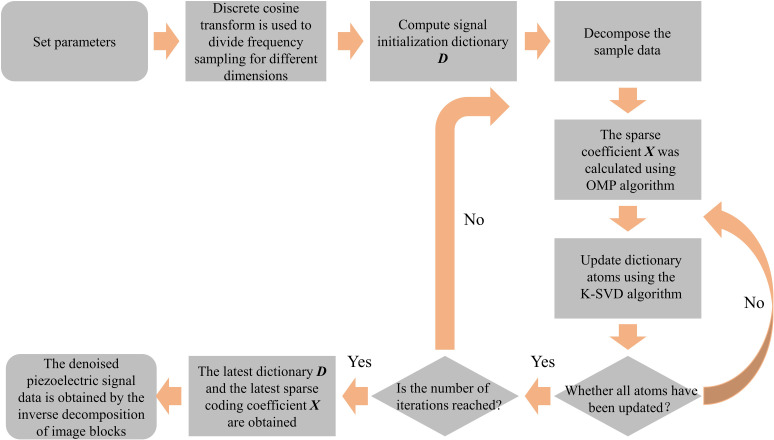
K-SVD dictionary learning process.

By decomposing the error matrix to obtain *U*, and updating $d_{k}$ in the dictionary ***D***. The sparse coefficient vector $x_{k}$ is updated by using $\Delta V^{T}$. The dictionary is continuously updated until the sparse decomposition error reaches a predetermined threshold, at which point iteration is stopped and the signal is output. The K-SVD dictionary learning algorithm processing flow is shown in Fig 1.

### 2.2 Evaluation of AE signal denoising effect based on K-SVD dictionary learning

To verify the feasibility and effectiveness of AE signal filtering technology based on K-SVD dictionary learning, a comparative analysis was conducted on the time-domain waveforms and parameters, as well as the frequency-domain parameters of the AE signal after K-SVD filtering and wavelet packet filtering. In 1985, Mitraković et al. [38] proposed a mathematical model describing simulated AE signals, with the following expression:


$$f(t)=\sum_{i=1}^{n}A_{i}e^{\left[-Q_{i}{\left(t-t_{i}\right)}^{2}\right]}\sin(2\pi f_{i}(t-t_{i}))$$ (6)

Where, $A_{i}$ represents the amplitude of the *i*-th superimposed signal. $Q_{i}$ represents the attenuation factor of the *i*-th superimposed signal. $f_{i}$ represents the dominant frequency of the *i*-th superimposed signal. $t_{i}$ represents the delay time of the *i*-th superimposed signal. *n* represents the number of superimposed signals in the simulated AE signal. The parameters selected for simulating AE signals in this study are as follows: n=3, $A_{1}=A_{2}={\text{A}}_{3}=2$, $Q_{1}=6.14\times 10^{8}$, $Q_{2}=1.48\times 10^{8}$, $Q_{3}=3.45\times 10^{8}$, $t_{1}=7\times 10^{-4}s$, $t_{2}=5\times 10^{-4}s$, $t_{2}=6\times 10^{-4}s$, $f_{1}=60\text{kHz}$, $f_{2}=90\text{kHz}$, $f_{3}=70\text{kHz}$. The sampling rate of the simulated AE signal is set to 1MSPS. During the AE test, various background noises interfere with the collected AE signals, often complicating the identification of critical features associated with crack initiation and growth. To realistically simulate the AE signals as they would be captured during actual testing, a controlled 10 dB white noise signal was added to the simulated AE data in the numerical simulation. This addition aimed to mimic the environmental noise encountered in field applications, thus providing a more accurate representation of real-world conditions and enabling an assessment of the K-SVD algorithm’s denoising performance.

**Fig 2:**
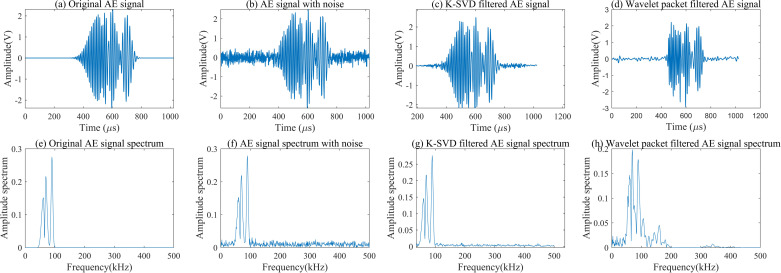
Comparison of AE signal time-frequency waveform under different conditions.

The AE signal simulation involved generating transient signals that reflect the expected characteristics of micro-crack formation and propagation, including high-frequency components indicative of crack-related stress waves. The added white noise, which has a flat spectral density across frequencies, ensures that the simulated noise closely resembles random environmental interference encountered in real scenarios. To evaluate the denoising effectiveness of the K-SVD dictionary learning algorithm, we applied the fast Fourier transform (FFT) to analyze and compare the spectral characteristics of three signal variations: the original simulated AE signal, the noisy AE signal, and the filtered AE signal processed by K-SVD. This spectral comparison, as illustrated in Fig 2, highlights the algorithm’s ability to suppress noise while preserving critical frequency components associated with AE events. This process allowed for a clear demonstration of how K-SVD enhances signal fidelity by selectively filtering out background noise, thereby improving the accuracy of subsequent feature extraction and classification tasks.

In this experiment, wavelet packet filtering serves as a benchmark for evaluating the performance of the K-SVD dictionary learning algorithm. Although wavelet packet filtering is effective for multi-scale denoising, it relies on predefined basis functions, limiting its adaptability to variations in AE signal characteristics under different damage states. In contrast, K-SVD dynamically learns a dictionary tailored to the specific features of AE signals, allowing for more flexible and adaptive denoising. By comparing these two methods, this study aims to highlight the strengths of K-SVD in providing robust noise suppression for AE signals, thereby enhancing its potential for real-time crack detection in steel bridge decks.

To quantify the denoising effect of K-SVD dictionary learning on AE signals, this study uses signal to noise ratio (*SNR*) and root mean square error (*RMSE*) to evaluate the denoising effect. When evaluating the denoising effect on AE signals, a higher signal-to-noise ratio and a smaller root mean square error between the denoised signal and the initial AE signal indicate better denoising performance of the filtering algorithm, as shown in Table 1.

**Table 1:** Evaluation of denoising effect.

Evaluation index	K-S VD	Wavelet packet
*SNR*	20.13 dB	15.24 dB
*RMSE*	0.0014	0.0283

As shown in Fig 2 and Table 2, K-SVD dictionary learning can effectively filter high-frequency noise in AE signals without causing distortion in the time-domain waveform of AE signals. The *SNR* of the denoised AE signal after K-SVD dictionary learning is 20.13dB and the *RMSE* is 0.0014. Wavelet packet denoising can cause distortion in the time-frequency waveform of AE signals, and the *SNR* of the denoised AE signal after wavelet packet denoising is 15.24dB and the *RMSE* is 0.0283. The denoising algorithm based on K-SVD dictionary learning can effectively filter environmental noise in AE signals and is more suitable for AE signal denoising.

**Table 2:** Fatigue crack identification accuracy of steel bridge panel.

Signal type	AE signals from undamaged areas		AE signals from damaged areas	
	Training accuracy	Test accuracy	Training accuracy	Test accuracy
Filtered AE signal	95.32%	94.27%	93.64%	92.56%
Unfiltered AE signal	83.27%	82.14%	85.36%	84.73%

## 3 Data acquisition and recognition principle

### 3.1 Test materials and protocols

**Fig 3:**
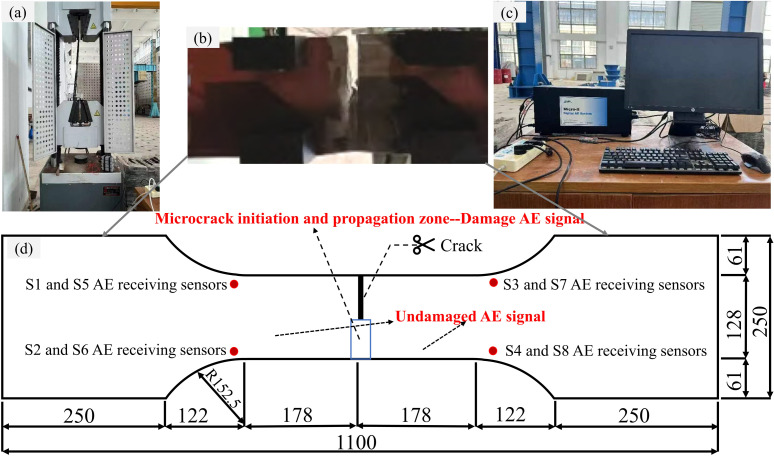
Test equipment. (a) 2000kN hydraulic servo fatigue testing machine, (b) Specimen, (c) PCI-II digital AE system, (d) Specimen model drawing.

To accurately identify the fatigue micro-crack activity state of a steel bridge deck, it is essential to collect AE signals from both damaged and undamaged steel bridge decks. This study collects AE data through indoor experiments using Q690q steel specimens with a thickness of 24mm and dimensions shown in Fig 3. The wave velocity is 5982 m/s. A 2000kN hydraulic servo fatigue testing machine was used for fatigue testing. During the test, the PCI-II Digital AE System developed by Physical Acoustics Corporation was employed to collect relevant acoustic emission data. The AE signals were received using eight Nano30-type AE probes. A crack was prefabricated at the center of the specimen. During the fatigue loading test, AE signals associated with the initiation, propagation, and unstable fracture activities of micro-cracks at the crack tip were defined as damage signals, while other AE signals were defined as undamaged signals. The AE localization technique was used to calibrate the location of the AE source.

The position of the AE receiving sensor was polished using a grinder, and a layer of Vaseline was applied to both the AE receiving sensor and the surface of the test piece to ensure full coupling. The AE receiving sensor was then fixed in place using a magnetic holder. In order to eliminate the influence of test environment noise on this test, the threshold value of the AE acquisition system was set to 35dB. The gain of the preamplifier was set to 40dB. The sampling frequency was set to 1MSPS. The response center frequency of the AE receiving sensor was set to 60~1000kHz. The sampling length was set to 1k.

### 3.2 Basic principle of AE positioning

Among the many AE source localization algorithms, the Geiger localization algorithm is the most widely used in practical engineering. It mainly starts from an initial solution. The correction amount for each iteration using the least squares method is calculated. The correction amount to the previous optimal solution is added. A new optimal solution is generated. The optimal solution is then brought into the objective function. If the error function value is not greater than the accuracy, the iteration is terminated, and the solution is the global optimal solution. Otherwise, the iteration continues to correct the amount and seek a new optimal solution. The objective function of the Geiger localization algorithm is shown below:


$$f_{\min}=\sum_{i=1}^{n}{\left({\left[{\left(x-x_{i}\right)}^{2}+{\left(y-y_{i}\right)}^{2}+{\left(z-z_{i}\right)}^{2}\right]}^{1/2}/v_{p}-(t_{i}-t_{0})\right)}^{2}$$ (7)

Where, (x,y,z) represents the spatial coordinates of the AE source. $t_{0}$ represents the occurrence time of the AE source. $\left(x_{i},y_{i},z_{i}\right)$ represents the location of the *i*-th AE receiving sensor. $t_{i}$ represents the time for acoustic emission to propagate to the location of the *i*-th AE receiving sensor. *n* represents the number of AE receiving sensors. $v_{p}$ represents the AE propagation speed. $f_{\min}$ represents the error function, also known as the objective function.

The positional relationship between the AE receiving sensor and the AE source is shown in the following formula:


$${\left[{\left(x-x_{i}\right)}^{2}+{\left(y-y_{i}\right)}^{2}+{\left(z-z_{i}\right)}^{2}\right]}^{1/2}\text{=}\nu_{p}(t_{i}-t_{0})$$ (8)

Taylor expansion of the above formula yields:


$$\begin{array}{c} t_{i}=t_{0}+t_{0i}^{'}+\frac{\partial t_{i}^{'}}{\partial x}\Delta x\text{+}\frac{\partial t_{i}^{'}}{\partial y}\Delta y+\frac{\partial t_{i}^{'}}{\partial z}\Delta z+\\ \frac{1}{2!}{\left(\frac{\partial t_{i}^{'}}{\partial x}\Delta x\text{+}\frac{\partial t_{i}^{'}}{\partial y}\Delta y+\frac{\partial t_{i}^{'}}{\partial z}\Delta z\right)}^{2}\\ +\frac{1}{n!}{\left(\frac{\partial t_{i}^{'}}{\partial x}\Delta x\text{+}\frac{\partial t_{i}^{'}}{\partial y}\Delta y+\frac{\partial t_{i}^{'}}{\partial z}\Delta z\right)}^{n} \end{array}$$ (9)

Where, $t_{0i}^{'}$ represents the travel time of the AE source propagating to the *i*-th AE receiving sensor. $t_{i}$ is the time when the P-wave propagates to the *i*-th AE receiving sensor position. Since the influence of the second order and higher order components on w is very small, within the allowable range of parameter reading errors, they can be ignored. Equation (9) can be simplified to the following equation, namely:


$$t_{i}=t_{0}+t_{0i}^{'}+\frac{\partial t_{i}^{'}}{\partial x}\Delta x\text{+}\frac{\partial t_{i}^{'}}{\partial y}\Delta y+\frac{\partial t_{i}^{'}}{\partial z}\Delta z$$ (10)

Among them, an auxiliary variable *R* is set, which is defined as follows:


$$R={\left[{\left(x-x_{i}\right)}^{2}+{\left(y-y_{i}\right)}^{2}+{\left(z-z_{i}\right)}^{2}\right]}^{1/2}$$ (11)

$$\frac{\partial t_{i}^{'}}{\partial x}=\frac{x_{i}-x}{v_{p}R}\;\frac{\partial t_{i}^{'}}{\partial y}=\frac{y_{i}-y}{v_{p}R}\;\frac{\partial t_{i}^{'}}{\partial z}=\frac{z_{i}-z}{v_{p}R}$$ (12)

*n* AE receiving sensors can obtain *n* equations as shown in equation (10), which can be simplified into a matrix form by combining them into a simultaneous equation system.


ΑΔθ=B (13)

$$\text{A}=\left[\begin{array}{l} 1\frac{\partial t_{1}^{'}}{\partial x}\frac{\partial t_{1}^{'}}{\partial y}\frac{\partial t_{1}^{'}}{\partial z}\\ 1\frac{\partial t_{2}^{'}}{\partial x}\frac{\partial t_{2}^{'}}{\partial y}\frac{\partial t_{2}^{'}}{\partial z}\\ \vdots \vdots \vdots \vdots \\ 1\frac{\partial t_{n}^{'}}{\partial x}\frac{\partial t_{n}^{'}}{\partial y}\frac{\partial t_{n}^{'}}{\partial z} \end{array}\right]\;\Delta \theta =\left[\begin{array}{l} \Delta t\\ \Delta x\\ \Delta y\\ \Delta z \end{array}\right]\;B=\left[\begin{array}{c} t_{1}-t_{0}\\ t_{2}-t_{0}\\ \vdots \\ t_{n}-t_{0} \end{array}\right]$$ (14)

Equation (13) is solved by using the least squares method.


$$\Delta \theta ={\left({\text{A}}^{T}\text{A}\right)}^{\text{-1}}{\text{A}}^{T}\text{B}$$ (15)

In the formula, Δθ represents the correction matrix. To determine the initial solution of the Geiger algorithm, the least squares method is used to locate the AE first to ensure that the initial solution of the Geiger algorithm is within the convergence region.

By establishing simultaneous equations for equation (8), we can obtain:


$${\left[{\left(x-x_{i}\right)}^{2}+{\left(y-y_{i}\right)}^{2}+{\left(z-z_{i}\right)}^{2}\right]}^{1/2}\text{=}\nu_{p}(t_{i}-t_{0})\;(i=1,2,\cdots ,n)$$ (16)

If one equation in the system of equations is the elimination term, and all other terms are simultaneously subtracted from the elimination term, then equation (16) can be simplified as shown below:


$$m_{i}x+h_{i}y+l_{i}z+f_{i}t=g_{i}\;(i=1,2,\cdots ,n-1)$$ (17)

Equation (17) can be written in matrix form as shown below:


CX=D (18)

$$\text{C}=\left[\begin{array}{l} m_{1}h_{1}l_{1}f_{1}\\ m_{2}h_{2}l_{2}f_{2}\\ \vdots \vdots \vdots \vdots \\ m_{n-1}h_{n-1}l_{n-1}f_{n-1} \end{array}\right]\;X=\left[\begin{array}{l} x\\ y\\ z\\ t \end{array}\right]\;D=\left[\begin{array}{c} g_{1}\\ g_{2}\\ \vdots \\ g_{n-1} \end{array}\right]$$ (19)

Solving equation (18) using the least squares method yields:


$$\text{X}={\left({\text{C}}^{T}\text{C}\right)}^{\text{-1}}{\text{C}}^{T}\text{D}$$ (20)

The solution obtained by formula (20) is the initial solution of the Geiger algorithm. Add the correction to the previous optimal solution to generate a new optimal solution. The optimal solution is substituted into the objective function. If the objective function value is less than or equal to the accuracy, terminate the iteration. This optimal solution is the global optimal solution. Otherwise, continue to iterate the correction and continue to seek new optimal solutions.

### 3.3 Principle of fatigue micro-crack detection based on convolutional neural network

A typical convolutional neural network mainly consists of two parts. One is the feature extractor, and the other is the classifier. The feature extractor consists of a module consisting of a convolutional layer and a pooling layer, while the classifier consists of fully connected layers. The convolutional layer is the feature extractor, which can extract local features of the input data. The convolution kernel slides over the input data sequence to calculate the linear weighted sum of local regions of the input data, forming a feature map. Each feature map can reflect the local patterns in the input data. Within the same local receptive field, different feature maps can be extracted by different convolution kernels, as shown in the following expression:


$$x_{i}^{l}=f(x_{i}^{l-1}*w_{i}^{l}+b_{i}^{l})$$ (21)

In the formula, $x_{i}^{l}$ represents the *i*-th feature map of the *l*-th layer of the input data. $x_{i}^{l-1}$ represents the output of the previous layer in the convolutional layer. $w_{i}^{l}$ represents the convolution kernel. $b_{i}^{l}$ represents the bias term of the convolutional layer.  *  represents the convolution operation. *f* represents the activation function of the convolutional layer.

The rectified linear unit has an activating effect on the units of the convolutional neural network. The rectified linear unit enables the units to perform linear calculations, which makes it easier for the model to undergo optimization operations. The pooling layer is usually used to reduce the dimensionality of the input feature map, thereby reducing the network model parameters and computational load.

To enhance the effectiveness of CNN in detecting fatigue cracks, this study integrates K-SVD as a denoising step that significantly improves CNN’s feature extraction capabilities. K-SVD is particularly effective for extracting key features from non-stationary signals by constructing an adaptive dictionary that accurately represents the underlying structure of AE signals while suppressing background noise. This preprocessing step refines the signal input to the CNN, allowing it to focus on distinct structural features associated with damage, thus enhancing classification accuracy.

In the context of signal processing, optimizing the integration of K-SVD and CNN involves tuning both components to work synergistically. First, selecting appropriate parameters for K-SVD—such as dictionary size and sparsity constraints—ensures that relevant features are preserved without over-smoothing. This refined signal is then fed into the CNN, where feature extraction layers are designed to capture subtle variations in AE signals indicative of damage progression. Additionally, an iterative feedback mechanism can be implemented to fine-tune the K-SVD parameters based on CNN performance metrics, ensuring that the denoising process continuously adapts to optimize CNN’s detection accuracy. Such integration of K-SVD and CNN can create a highly efficient signal processing chain, balancing computational complexity with real-time application potential, and making this approach viable for practical, automated bridge inspection.

### 3.4 Model parameter setting and model identification effect evaluation method

In order to evaluate the accuracy of the fatigue micro-crack identification of steel bridge deck based on the CNN model, this study selects the root mean square error (*RMSE*), mean absolute error (*MAE*), and coefficient of determination (*R*^2^) between the predicted value and the measured value to evaluate the model. The definitions of *RMSE*, *MAE*, and *R*^2^ are as follows:


$$RMSE\text{=}\sqrt{\frac{1}{m}\sum_{i=1}^{m}{\left(f(x_{i})-y_{i}\right)}^{2}}$$ (22)

$$MAE=\left|\frac{1}{m}\sum_{i=1}^{m}\left(f(x_{i})-y_{i}\right)\right|$$ (23)

$$R^{2}=1-\frac{\sum_{i=1}^{m}{\left(f(x_{i})-y_{i}\right)}^{2}}{\sum_{i=1}^{m}{\left(\bar{y}-y_{i}\right)}^{2}}$$ (24)

Where, *m* represents the total number of samples in the prediction data set. $f(x_{i})$ represents the prediction data of the CNN model. $y_{i}$ represents the category of the actual AE signal. $\bar{y}$ represents the mean value of the category of the measured AE signal. The coefficient of determination is used to evaluate the correlation between the model and the prediction data. Its value range is distributed in the interval of 0–1. A larger the *R*^2^ value indicates a better the prediction effect of the model.

Before training the CNN model, it is common to use cross-validation to determine the optimal parameters for the model. The model parameters mainly include the number of neurons in the convolutional layer, the number of layers in the neural network, the learning rate, the optimization function, the dropout rate, the number of iterations, and the batch size. The cross-validation algorithm randomly selects 20% of the data as a validation set, and trains the remaining 80% of the data under different parameter combinations. The parameter combination with the smallest root mean square error is selected as the optimal parameter combination for the model.

## 4 Analysis and discussion of test results

### 4.1 Evolution characteristics of AE during the damage and failure process of cracked steel plates under fatigue loading conditions

**Fig 4:**
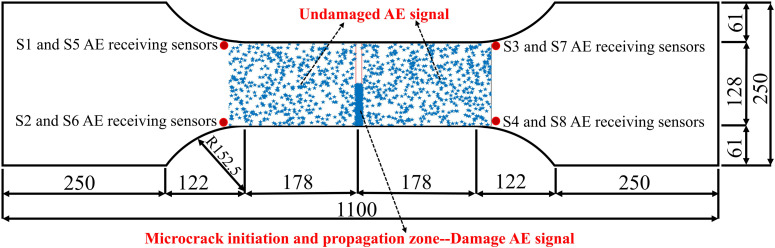
AE positioning projection.

In order to calibrate the damage and AE signals from undamaged areas associated with the damage and failure process of cracked steel plates under fatigue loading conditions, this study uses AE localization technology to quantify the distribution location of AE sources. The results are shown in Fig 4. The AE source localization was conducted using the Geiger localization algorithm, with a longitudinal wave velocity of 5982 m/s determined for the steel bridge panel material. This velocity was used in the time difference of arrival (TDOA) calculations to enhance localization accuracy. AE localization technology is used to study the spatial distribution characteristics of AE sources. It is necessary to ensure that at least four AE receiving sensors can receive information from the same AE source. A total of 1473 sets of locatable AE signals were generated during the fatigue loading test of cracked steel plates, including 389 sets of locatable AE signals generated in the microcrack initiation and propagation region, and 1084 sets of locatable AE signals generated in the undamaged region due to the sliding friction of crystal particles.

As shown in Fig 4, under fatigue loading conditions, a large number of AE signals are generated in the undamaged area during the damage and failure process of the cracked steel plate. The S1-S8 AE receiving sensors collected a total of 6,721 AE signals from undamaged areas, in addition to 2,913 AE signals from damaged areas. The core work of steel bridge fatigue detection research is to find data related to micro-crack initiation and propagation from massive detection data. The AE signals generated in the undamaged area pose a great challenge to steel bridge detection work.

### 4.2 Time-frequency characteristics of AE signals from undamaged and damaged areas

**Fig 5:**
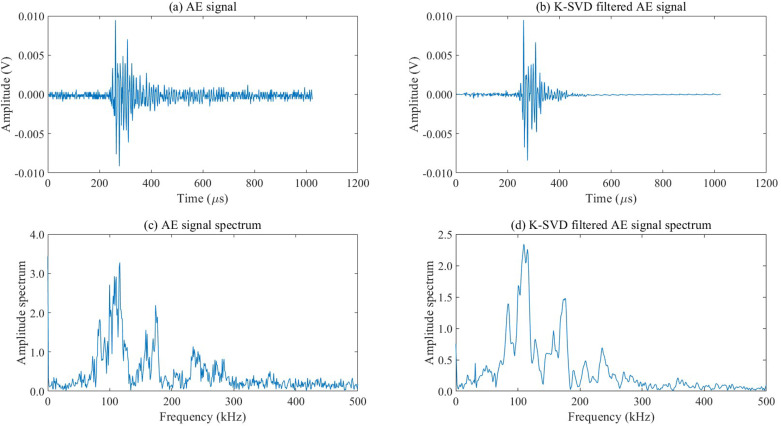
Time-domain and frequency-domain waveform diagrams of AE signals collected from undamaged areas.

**Fig 6:**
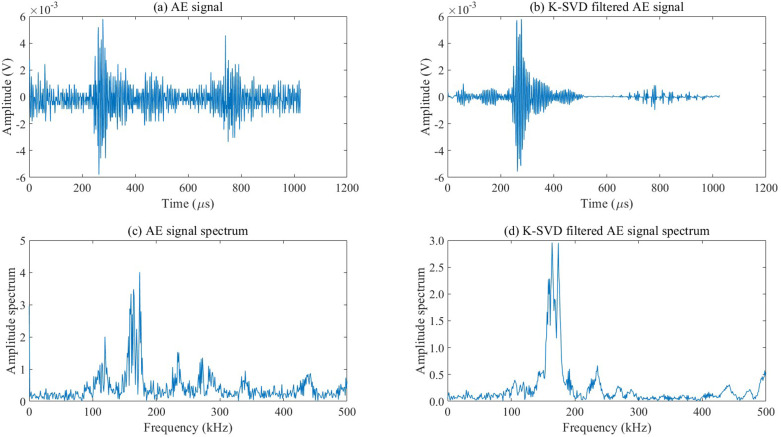
Time-domain and frequency-domain waveform diagrams of AE signals collected from the damaged area.

As a new dynamic monitoring and detection technology, AE has been widely applied in the field of bridge health assessment. By collecting transient elastic stress waves released rapidly from the interior of materials under load, it can achieve dynamic assessment of bridge damage status. This study uses the K-SVD dictionary learning algorithm to filter the AE signals collected from damaged and undamaged areas, and uses the fast Fourier transform to analyze the frequency domain characteristics of the filtered AE signals, as shown in Figs 5 and 6.

As illustrated in Figs 5 and 6, the K-SVD dictionary learning filtering algorithm effectively suppresses background noise in both damaged and AE signals from undamaged areas, demonstrating good filtering effects on both types of signals. This indicates that the K-SVD dictionary learning filtering algorithm possesses strong adaptive representation capabilities, allowing it to adaptively learn a dictionary from AE signals of steel structures in various damage states. It can accurately represent AE signals based on the structure and characteristics of the basic functions.

The maximum amplitude of the time-domain waveform of AE signals from undamaged areas is greater than that of AE signals from damaged areas because the energy decay rate of AE signal propagation is lower in undamaged areas compared to damaged areas. The time-domain waveforms of AE signals from undamaged areas have relatively clean first and tail waves, while the damaged area contains numerous microcracks. As AE signals pass through the microcrack distribution area, they are prone to physical phenomena such as reflection and diffraction, resulting in complex superimposed waves. The dominant frequency of AE signals from damaged areas is 185 kHz, whereas that of AE signals from undamaged areas is 116 kHz.

### 4.3 Fatigue micro-crack identification of steel bridge deck based on CNN

AE signals are non-stationary random signals, often mixed with various background noises in practical engineering applications. Due to the random distribution characteristics of noise throughout the sampling period, K-SVD dictionary learning denoising cannot completely eliminate it. Therefore, it is essential to analyze both the time-domain and frequency-domain characteristics of the AE signal, extracting the time-frequency information features to achieve intelligent identification of fatigue micro-cracks in steel bridge panels.

To address the challenge of identifying fatigue damage in steel structures based on AE signals, this paper employs SVD technology to decompose the time-frequency waveform of filtered AE signals. This approach constructs feature matrices of the time-frequency waveform of AE signals under different damage states in steel structures. A CNN model with normalized learning capabilities is used to identify fatigue cracks in steel bridge panels.

Using AE localization technology, AE signals collected during the experiment are classified into damage-type and undamaged-type categories. The K-SVD dictionary learning filter and SVD algorithm are employed to filter, extract features, and normalize the input data of the AE signals obtained during the experiment, providing input feature matrices for the CNN fatigue micro-crack identification model. All collected AE signals are randomly divided into two parts, with 80% as training data and 20% as testing data, to realize the identification of fatigue cracks in steel bridge panels.

The aforementioned data preprocessing methods were used to preprocess the AE signal monitoring data and to train a CNN network model. The CNN network model was built using the TensorFlow toolkit and implemented with Python. The training hardware for the CNN model was a Lenovo P620 workstation, equipped with an AMD CPU with 64 cores, 128 GB of RAM, and an RTX 4090 GPU.

The CNN network model employed a batch training approach, with each batch containing 128 AE signal feature matrices. The maximum number of iterations was set to 80. The input data dimensions were 32×32, and the output data dimensions were 2×1, representing damaged and non-damaged signal classifications. The model was configured with multiple convolutional layers to capture hierarchical features from the AE signals. The convolutional layers used a 3×3 kernel size to extract localized features, followed by ReLU activation functions to introduce non-linearity.

Pooling layers were applied after each convolutional layer to reduce spatial dimensions, with max pooling used to retain the most prominent features while minimizing information loss. These pooling layers help in enhancing computational efficiency and in preventing overfitting by down sampling the feature maps. After several convolutional and pooling layers, the data was flattened and passed through fully connected layers to integrate and classify the learned features.

The Adam optimizer was used, with a weight decay of 0.005 and a learning rate of 0.01, ensuring efficient convergence. The objective function was constructed using a cross-entropy loss function, which is well-suited for binary classification. To validate the role of the K-SVD dictionary learning filtering algorithm in identifying fatigue cracks in steel bridge panels, crack identification tests were conducted on both the filtered AE signals and the raw collected AE signals. The results, presented in Table 2, demonstrate the effectiveness of the CNN model combined with K-SVD filtering in improving the accuracy of fatigue crack identification in steel bridge panels.

Table 2 demonstrates that the AE signals filtered by the K-SVD dictionary learning method achieved high accuracy in the CNN network model’s training and testing phases. Specifically, the recognition accuracy for AE signals from damaged areas in the training and test sets was 93.64% and 92.56%, respectively. For AE signals from undamaged areas, the recognition accuracy was 95.32% and 94.27% in the training and test sets, respectively. The presence of noise in the AE signals increased the difficulty for the CNN network model to extract the input matrix features, leading to suboptimal recognition accuracy in both the training and test sets. The recognition accuracy for AE signals from damaged areas in the noisy training and test sets was 85.36% and 84.73%, respectively, while for AE signals from undamaged areas, it was 83.27% and 82.14%, respectively. This is because noise can obscure the information about crack initiation and propagation within the AE signals. The K-SVD dictionary learning filtering algorithm effectively reduces the interference of background noise in AE signals. Singular value decomposition extracts the time-frequency feature input matrices of AE signals, which can visually represent the fatigue crack initiation mechanism. The CNN network model, leveraging its strong visual learning capabilities, successfully extracted the features of AE signals from damaged areas, improving accuracy and robustness. Therefore, AE signals collected from damaged and undamaged regions during fatigue damage tests on steel structures, combined with the filtering capability of the K-SVD dictionary learning and the feature extraction capability of the CNN network, can achieve intelligent identification and classification of fatigue cracks in steel bridge panels.

To validate the recognition performance of the CNN model, this study also applied BP neural networks, Naive Bayes, and Random Forests to process the experimental data. The *RMSE*, *MAE*, and R² of the test sets were statistically analyzed, as shown in Table 3.

**Table 3:** Evaluation of performance of different models in fatigue crack type identification.

Parameters	BP Neural Network	Naive Bayes	Random Forest	CNN
*RMSE*	0.3777	0.2975	0.2500	0.1484
*MAE*	0.3005	0.2428	0.2064	0.1205
*R* ^2^	0.8368	0.8650	0.8729	0.9356

Table 3 indicates that the BP neural network exhibited the largest error in fatigue crack identification, with *RMSE*, *MAE*, and *R*² values of 0.3777, 0.3005, and 0.8368, respectively, in the test set. The Naive Bayes algorithm achieved *RMSE*, *MAE*, and *R*² values of 0.2975, 0.2428, and 0.8650, respectively. The Random Forest algorithm showed *RMSE*, *MAE*, and *R*² values of 0.2500, 0.2064, and 0.8729, respectively. The CNN model had the smallest error, with *RMSE*, *MAE*, and *R*² values of 0.1484, 0.1205, and 0.9356, respectively, in the test set. The slight discrepancies between training and test accuracies can be attributed to several factors. First, the test set represents new data that the model has not previously encountered, meaning minor variations in AE signal characteristics could lead to slightly lower performance as the model adjusts to these differences. Additionally, the K-SVD filtering and CNN classification steps may face challenges with subtle signal variations, especially if the noise in the test set differs in pattern or intensity from the training set. In summary, the CNN network model, using time-frequency waveform features of AE signals as input, demonstrates superior performance in identifying fatigue cracks in steel bridge panels. The research findings offer a novel detection method for monitoring fatigue cracks in steel bridge panels.

The findings of this study have significant implications for real-world bridge health monitoring systems, particularly in advancing the early detection of fatigue cracks in steel bridge panels. The high-accuracy classification of damaged versus AE signals from undamaged areas achieved here demonstrates that an AE-based system utilizing K-SVD filtering and CNN classification can provide a reliable tool for assessing structural health. This capability enables proactive maintenance interventions, potentially preventing catastrophic failures and extending the operational lifespan of bridges.

However, several limitations must be addressed before field deployment. One key challenge is the computational cost associated with the K-SVD algorithm, which, while effective in filtering out noise and improving signal clarity, can be resource-intensive for real-time processing, especially on large-scale bridge structures. For practical applications, optimized or alternative algorithms with lower computational demands may be explored. Additionally, the model’s applicability across various bridge types remains to be validated. Differences in structural materials, design, and environmental conditions, such as temperature and humidity, could impact AE signal characteristics, and the model may require retraining or further adaptation to achieve similar accuracy levels in diverse contexts.

Future work will focus on refining and optimizing this approach to meet the demands of continuous, real-time bridge monitoring. This includes exploring alternative filtering techniques or lightweight variations of the K-SVD algorithm to reduce computational load, as well as expanding training datasets to include AE signals from different bridge designs, materials, and environments to enhance generalization. Field tests on operational bridges will be essential for assessing system robustness in situ, enabling further adjustments and enhancements for reliable performance under diverse environmental and structural conditions. Additionally, integrating this system with existing bridge monitoring frameworks, such as vibration monitoring or structural deformation sensors, could create a comprehensive solution for real-time, multi-faceted health monitoring, paving the way for safer and more efficient infrastructure management.

## 5 Conclusions

(1) This study addresses the non-stationarity of AE signals during the initiation and propagation of cracks in steel bridge panels. By employing the K-SVD dictionary learning algorithm for filtering AE signals and combining it with AE localization technology to calibrate the AE signal generation mechanism, a novel high-precision method for identifying fatigue cracks in steel bridge panels is proposed.
(2) The K-SVD dictionary learning filtering algorithm effectively suppresses background noise in both damaged and AE signals from undamaged areas, demonstrating good filtering performance for both types. This indicates that the K-SVD dictionary learning filtering algorithm has strong adaptive representation capabilities, allowing it to learn dictionaries adaptively from AE signals of steel structures in different damage states. It accurately represents AE signals under various damage conditions based on the structure and features of the basic functions.
(3) The maximum amplitude of the time-domain waveform of AE signals from undamaged areas is greater than that of AE signals from damaged areas. The time-domain waveform of AE signals from undamaged areas has relatively clean first and last waves, whereas the damaged region contains numerous microcracks. As AE signals pass through the microcrack distribution area, they are prone to reflection, diffraction, and other physical phenomena, resulting in complex superimposed waves. The main frequency of AE signal from damaged areas is 185 kHz, while that of AE signal from undamaged areas is 116 kHz.
(4) AE signals filtered by the K-SVD dictionary learning algorithm achieve high accuracy in the training and testing phases of the CNN network model. The recognition accuracy for AE signals from damaged areas in the training and test sets is 93.64% and 92.56%, respectively, and for AE signals from undamaged areas, it is 95.32% and 94.27%, respectively.
(5) The practical application of this method in bridge monitoring systems could significantly enhance maintenance strategies by enabling early crack detection and facilitating timely interventions, which would reduce maintenance costs and extend bridge service life. This intelligent monitoring approach would be invaluable for large-scale infrastructure, where continuous assessment of structural health is critical to ensuring safety and functionality. Future work will focus on optimizing this system for diverse bridge types and environmental conditions, further enhancing its practicality and impact on transportation safety and infrastructure management.
